# Acidity of the Stomach

**Published:** 1893-04

**Authors:** 


### Acidity of the Stomach.

This is not unfrequently curable by lemon juice or citric acid, after all kinds of antacids have been tried in vain. The explanation is this: A depraved state of the mucous membrane lining the stomach, dependent on loss of tone, is one of the sources of acidity; and this acidity is often subdued by the tonic action found in the lemon acid or juice.—*Med. Summary.*

